# Quantitative Analyses of Nine Phenolic Compounds and Their Antioxidant Activities from Thirty-Seven Varieties of Raspberry Grown in the Qinghai-Tibetan Plateau Region

**DOI:** 10.3390/molecules24213932

**Published:** 2019-10-31

**Authors:** Yuwei Wang, Jian Liang, Guangxiang Luan, Shoude Zhang, Yixi Zhuoma, Jiuxiang Xie, Wu Zhou

**Affiliations:** 1 State Key Laboratory of Plateau Ecology and Agriculture, Qinghai University, Xining 810016, China; wangyuwei0507@163.com (Y.W.); liangjianws@126.com (J.L.); shoude.zhang@foxmail.com (S.Z.); yixizhuoma8899@126.com (Y.Z.); xiejiuxiang8817@163.com (J.X.); 2 Key Laboratory of Tibetan Medicine Research, Northwest Institute of Plateau Biology, Chinese Academy of Sciences, Xining 810016, China; gxluan@nwipb.cas.cn

**Keywords:** RP-HPLC/UV, raspberry, phenols, RSM, antioxidant activity

## Abstract

In this work, an efficient method for the rapid extraction and separation of antioxidant phenols was developed and optimized. The method was then applied to extract and separate nine phenols from 37 varieties of raspberry, in which their antioxidant activities were further investigated. First, the extraction was conducted using ultra-sonication, which was then further separated using reversed-phase high-performance liquid chromatography/ultraviolet (RP-HPLC/UV) analysis. In this step, several key parameters (volume of the extraction reagent, time of extraction, and the temperature of extraction) affecting its efficiency were investigated and optimized using the response surface methodology (RSM) combined with the Box–Behnken design (BBD) so that the optimal conditions were obtained. According to the overall results of the optimization study, the optimal conditions were chosen as follows: volume of extraction reagent = 2.0 mL, time of extraction = 50.0 min, and temperature of extraction = 50 °C. The optimal conditions were then applied to extract nine phenols, including gallic acid, catechin, chlorogenic acid, vanillic acid, syringic acid, cumaric acid, ferulic acid, rosemary acid, and quercetin from 37 raspberry varieties. The extracted phenols were characterized and their antioxidant activities, including DPPH^−^ and ABTS^−^ free radical scavenging and intracellular reactive oxygen species (ROS) activity, using HepG2 cells as the model, were subsequently studied. The findings suggested that although their contents varied among most raspberry varieties, these phenols significantly contributed toward their antioxidant capacity and scavenging intracellular ROS activities. This study provides a scientific and theoretical basis for the selection of raspberry varieties and product development in Qinghai province.

## 1. Introduction

Raspberry (*Rubus corchorifolius L. f*) belongs to the family Rosaceae, the subfamily Rosoideae, and the genera *Rubus*. The plant has high levels of flavonoids [1,2], anthocyanins [3,4], phenols [5], vitamin C, dietary fibers, tocotrienol, calcium, magnesium, linoleic acid, and carotenoids [6].

Phenols, the phenylpropanoid compounds derived from phenylalanine and the tyrosine metabolic pathways, are active, natural antioxidant compounds found in various plants [7]. Various studies have shown that phenols play important roles as antioxidants against free radicals and other reactive oxygen species (ROS), which are the major cause of many chronic human diseases, such as cancer and cardiovascular diseases [8,9,10]. Moreover, phenols from the cranberry plant and its products have been shown to have rich biological activities involving anticarcinogenic [11], antimutagenic [12], antibacterial [13], antioxidant, and antiradical properties [14], which are evident from various in vitro and animal model studies. In addition, some of the phenol derivatives, such as ferulic acid, caffeic, and p-coumaric acid, have been described to be important functional compounds that contain decent antioxidant activities [8]. Furthermore, as one of the most abundant phenol derivatives, rosmarinic acid has been extensively utilized in the pharmaceutical and cosmetic industries because it has a high antioxidant activity [15]. Despite the above, bioactive compounds derived from berries are still rarely identified and extracted because of their low content, and the complex composition of berries leads to a high content of other compounds that interfere with extraction and separation. 

**Figure 1:**
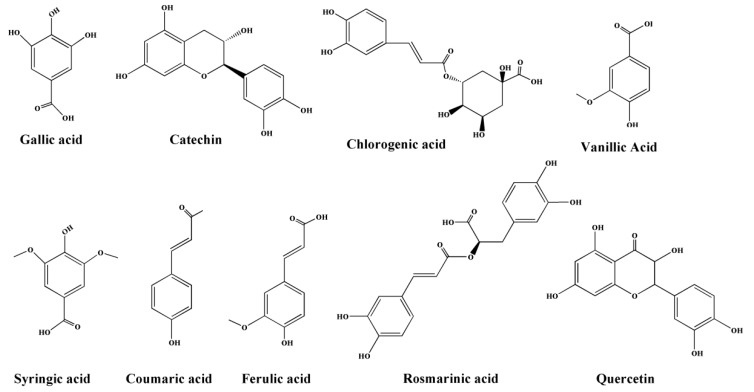
The chemical structures of nine phenols.

Because of their structures (Figure 1), phenols have a limited absorbance selectivity in the ultraviolet region. Because of this property, the most commonly used method for the determination of phenolic compounds is based on high-performance liquid chromatography (HPLC) separation [16,17]. High-performance liquid chromatography-electrospray ionization-mass spectrometry (HPLC-ESI-MS) [18], high-performance liquid chromatography–diode array detection-electrospray ionization-mass spectrometry (HPLC-DAD-ESI-MS) [19], and high-resolution MS [20] can be used to determine the total phenol content, which provides information on the molecular mass and structural features of components, and they are considered to be more useful than other methods of separation, identification, and quantification of the characteristic compounds. However, since these methods are quite costly when achieving short analysis times, or long analysis times with common equipment, many studies [21,22,23] have used high-performance liquid chromatography/ultraviolet (HPLC/UV) detection, which is less costly, decreases the runtime, is comparably convenient to operate, minimizes wear on various HPLC system components, and is suitable for routine analysis for the determination of phenolic compounds. Studies describing the methodology for determining phenolic compounds in raspberry plants grown in the Qinghai-Tibetan Plateau region using reversed-phase high-performance liquid chromatography/ultraviolet (RP-HPLC/UV) have not been found. Extraction is the initial process, and it is also an essential step in the recovery and purification of phenolic compounds from raspberries. The efficiency of extraction depends on several key parameters, such as the volume of the extraction reagent, time of extraction, the temperature of extraction, and pH, and their effects can be either independent or interactive. In this study, the response surface methodology (RSM) combined with the Box–Behnken design (BBD) was used to statistically evaluate the effects of multiple factors and their interactions. Therefore, it is of great importance that sensitive and accurate extraction and separation methods of phenolic compounds from berries are established. 

Raspberry planting is a labor-intensive industry with high labor costs. In recent years, raspberry production has been stagnant or has shrunk in developed countries, such as those in Europe and the United States, and many western European countries are withdrawing from the raspberry production field. Therefore, the demand gap for raspberries is constantly expanding. At present, raspberry planting is growing rapidly in some developing countries. Thirty-seven varieties of raspberry are grown in the Qinghai-Tibetan plateau region, including 9 local varieties and 28 adventitious varieties. In this work, an efficient method for the rapid extraction and separation of phenols was developed, and several key parameters affecting its efficiency were optimized using a Box–Behnken design (BBD) in accordance with the response surface methodology (RSM). The optimized methods were then applied to an analysis of 9 phenols including gallic acid, catechin, chlorogenic acid, vanillic acid, syringic acid, cumaric acid, ferulic acid, rosemary acid, and quercetin from 37 varieties of raspberry grown in the Qinghai-Tibetan Plateau region. The raspberry-extracted phenols were further tested for their antioxidant activities, including scavenging activities against 1,1-diphynyl-2-picrylhydrazyl (DPPH) and 2,2′-azino-bis(3-ethylbenzothiazoline-6-sulfuric acid) (ABTS) free radicals, as well as intracellular reactive oxygen species (ROS) using HepG2 cells as the model. To the best of our knowledge, this is the first reported extraction, separation, and analysis of phenols and their antioxidative activities from 37 varieties of raspberry grown in the Qinghai-Tibetan Plateau region, and this study provides a scientific basis for the selection of raspberry varieties and product development.

## 2. Results and Discussion

### 2.1. Optimization of the Extraction Conditions

In order to obtain the best extraction conditions, a BBD was employed to optimize the significant variables, as well as to further investigate interactions between these variables. The examined levels and experimental results are listed in Table 1. The large “model F-value” of 24.37 indicated that the model was significant, with the chance of only 0.02% that this might be due to noise. Moreover, the values of “prob > F” were lower than 0.0500, indicating that the model terms, which included A, C, AB, A^2^, B^2^, and C^2^, were significant. This means that the volume of extraction reagent, the temperature of extraction, interaction of the volume of extraction reagent, and the time of extraction were, indeed, key parameters affecting the extraction efficiency. The “lack of fit F-value” of 7.79 implied that it was significant, with a 3.80% probability that this occurred due to noise. The empirical second-order polynomial model for the extraction design is shown in the following equation:

(1) $$Y_{1}=+5154.02+28.02\times A+11.02\times B+26.71\times C+20.91\times AB+17.07\times AC\;-4.29\times BC-67.26A^{2}-46.32B^{2}-49.33C^{2}\;$$

**Figure 2:**
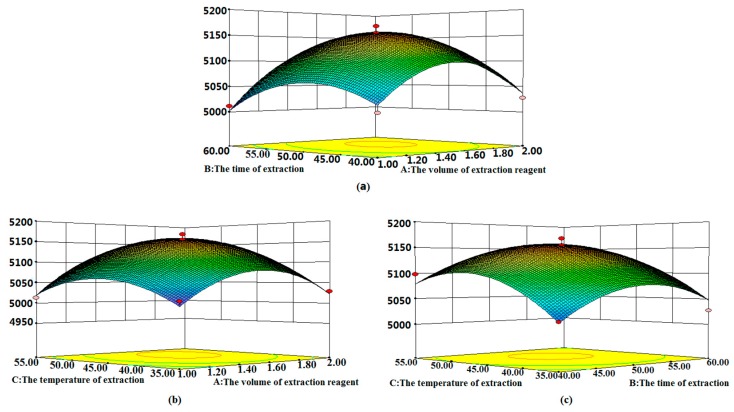
3D surface plot showing the significant interaction effects of the extraction parameters: (**a**) the volume of extraction reagent and the time of extraction, (**b**) the volume of extraction reagent and the temperature of extraction, and (**c**) the time of extraction and the temperature of extraction.

Based on the optimal conditions, three-dimensional response surfaces (Figure 2a–c) were plotted to investigate the interactions among the variables in order to determine the optimization of each factor for the maximum content of phenols. Figure 2a shows the combined effect of the volume of extraction reagent and the time of extraction. Figure 2b highlights the volume of extraction reagent and the temperature of extraction. Figure 2c depicts the combined effects of the time of extraction and the temperature of extraction. According to the overall results of the optimization study, the optimal conditions were chosen as follows: volume of extraction reagent = 2.0 mL, time of extraction = 50.0 min, and the temperature of extraction = 50 °C.

### 2.2. Optimization of HPLC Separation 

**Figure 3:**
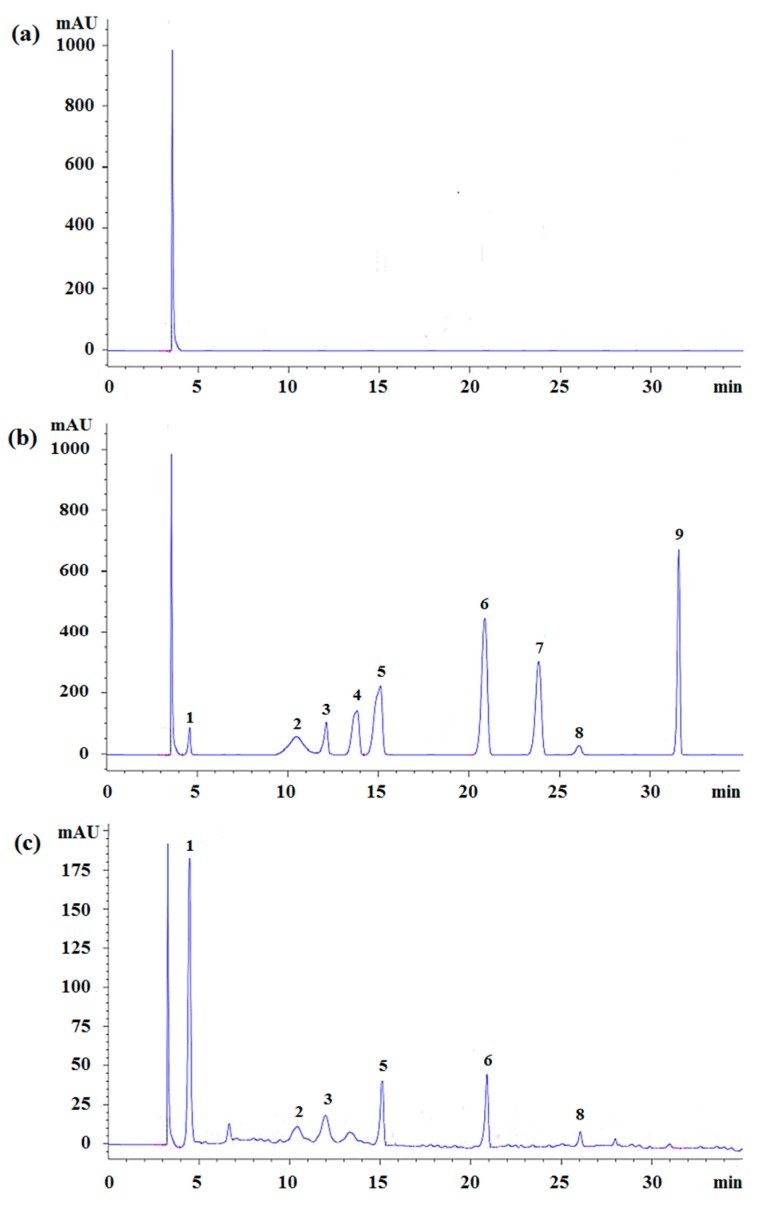
The representative chromatograms for blank (**a**), standards (**b**), and the typical chromatograms for the nine phenols in Canby (**c**). Peak labels: 1—gallic acid, 2—catechin, 3—chlorogenic acid, 4—vanillic acid, 5—syringic acid, 6—cumaric acid, 7—ferulic acid, 8—rosemary acid, and 9—quercetin.

To obtain the optimal HPLC separation conditions, several chromatographic parameters were investigated—chromatographic columns including Hypersil™ BDS C8 (200 mm × 4.6 mm, 5 μm, Thermo Fisher Scientific, Waltham, MA, USA), Agilent Zorbax C18 (250 mm × 4.6 mm, 5 μm, Agilent Technologies Co. Ltd., Palo Alto, CA, USA), Spherisorb^®^ C18 (200 mm × 4.6 mm, 5 μm, Waters, Milford, MA, USA), Zorbax Eclipse XDB-C8 (150 mm × 4.6 mm, 5 μm, Agilent Technologies Co. Ltd., Palo Alto, CA, USA), Hypersil™ GOLD (250 mm × 4.6 mm, 5 μm, Thermo Fisher Scientific, Waltham, MA, USA), and Hypersil C18 (200 mm × 4.6 mm, 5 μm, Thermo Fisher Scientific, Waltham, MA, USA), along with their separation efficiencies, were compared. The results indicated that the Hypersil™ GOLD (250 mm × 4.6 mm, 5 μm) column had the highest separation efficiency among all columns investigated. In the mobile phase, in which acetonitrile–water and methanol–water were compared, the results showed that a more symmetric separation peak was obtained when acetonitrile–water was used as the mobile phase. Based on the above results, the chromatographic conditions, which are considered optimal, were as follows: analytical column, Hypersil™ GOLD (250 mm × 4.6 mm, 5 μm) column; temperature, 30 °C; and mobile phase, acetonitrile–water. Other conditions were (1) elution conditions, including two types of eluents: eluent A (0.3% methanoic acid aqueous solution) and eluent B (5% acetonitrile and 0.3% methanoic acid aqueous solution); (2) flow steps of eluent B: 98%–95% from 0–8 min, 95%–89.5% from 8–18 min, 89.5%–89.5% from 18–21 min, 89.5%–75% from 21–30 min, and 75%–0% from 30–35 min; and (3) sample injection volume: 10 μL. In addition, prior to each analysis, the column was pre-equilibrated with the mobile phase for 5 min. The HPLC chromatograms of the blank sample, standard solutions, and the extracted samples are presented in Figure 3.

### 2.3. Validation of the Method

The optimized method was validated for its linearity, limits of detection (LODs), the limits of quantification (LOQs), precision, and accuracy. Linearity data were obtained by a plot of the peak areas versus concentrations for nine phenol standards. As summarized in Table 2, all phenols showed excellent linear responses with coefficients of >0.9962. In addition, the LOD and LOQ ranges were from 0.12 to 0.49 ng/mL and 0.35 to 1.02 ng/mL, respectively. The instrument precision determined based on phenols was lower than 1.1 and 1.4, respectively, for the inter-day and intra-day validations (Table 2). The percent recoveries were determined by comparing the concentrations obtained from spiked samples (conducted by spiking three different concentrations of samples into standards) with that of the actual sample amount added. As reported in Table 3, the percent recoveries ranged from 94.0% to 101.1%. These results demonstrated that this method was a precise and practical method, suitable for the determination of phenols extracted from the raspberries.

### 2.4. Analysis of 9 Phenols from 37 Raspberry Varieties 

The established method, as well as its optimal conditions, was further applied to analyze 9 phenols from 37 raspberry varieties: 28 adventitious varieties (Meeker, Boyne, Tulameen, Fortodi, Lauren, Canby, Taylor, Tulameen, Reveille, Coho, Encore, Herokee, Kitsilano, Chilcotin, Titan, Latham, Raspberry Nano, Chillieack, Triple Crown, Boysenberry, Shawnee, Honey Queen, Full of Red Raspberry, Autumn Britten, Autumn Bliss, Heritage, Killarney, and Kiwigold), and 9 local varieties (Laguo, Cangjia, Baojia, Leren, Nanque, Ganchong, Pansheng, Layun, and Huazang). The composition data of the nine phenols in the dry materials are expressed as mean ± SD (*n* = 3) and summarized in Table 4. The data showed that the contents of 9 phenols from 37 raspberry varieties were significantly different, in which the content from the adventitious varieties was higher than that from the local varieties. Moreover, the contents of gallic acid, catechin, chlorogenic acid, syringic acid, and cumaric acid were highest in the adventitious varieties of Tulameen (195.51 mg/g), Coho (59.69 mg/g), Shawnee (391.60 mg/g), Full of Red Raspberry (313.78 mg/g), and Meeker (198.21 mg/g). In addition, the contents of vanillic acid, ferulic acid, and quercetin acid were found to be very low. The content of rosemary acid was less 25.06 mg/g, except for Encore (38.55 mg/g) and Boysenberry (33.62 mg/g) varieties. 

The phenolic compound contents of the raspberries growing in different regions were significantly different [24,25]. The content of ferulic acid in raspberry cultivars grown in Turkey was 6.39 mg/g, which was higher than that of raspberry cultivars grown in the Qinghai–Tibetan Plateau region [24]. The content of quercetin in raspberry cultivars grown in Turkey was 0.35mg/g, and it was found that only Boyne contained quercetin among raspberry cultivars grown in the Qinghai–Tibetan Plateau region. The phenolic compositions of raspberries were quite different between those grown in Poland and the Qinghai–Tibetan Plateau region [25]. In addition, the amount of total phenolic compound in extracts was determined according to the Folin–Ciocalteu’s procedure in order to prepare with other studies concerning rasberries from other geographic places. As shown in Table 5, the total phenolic compound contents of Heritage and Meeker growing in Qinghai-Tibetan Plateau was much higher than Northern Greece, New York and so on. The total phenolic compound contents of Autumn Bliss in Qinghai-Tibetan Plateau was lower than Northern Greece but higher than Spain, Belgrade, Brazil, New York. The total phenolic compound contents of Taylor, Boysenberry, Kiwigold, Autumn Britten, Boyne, Tulameen, Coho planted in different areas were also significantly different. These variations in phenol content might be due to differences in physical and climatic environments, such as temperature, soil, moisture, wind, humidity, sunlight, and so on.

### 2.5. Analysis of Antioxidant Activities

#### 2.5.1. The DPPH and ABTS Free Radical Scavenging Activities

**Figure 4:**
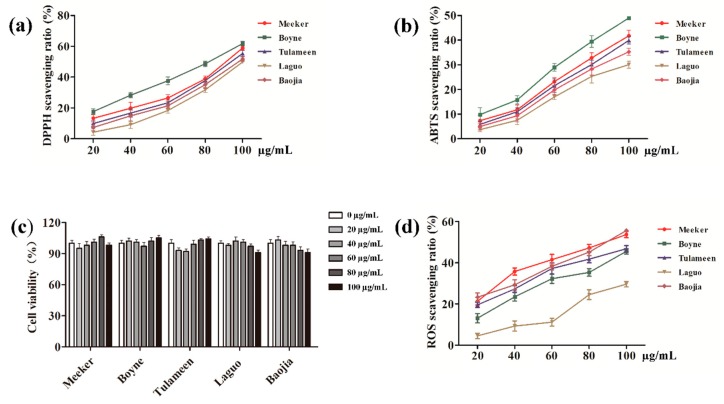
Analysis of antioxidant activities: (**a**) 1,1-diphenyl-2-picrylhydrazyl (DPPH) scavenging ratio, (**b**) 2, 2′-azino-bis(3-ethylbenzothiazoline-6-sulfonic acid) (ABTS) scavenging ratio, (**c**) cell viability, and (**d**) reactive oxygen species (ROS) scavenging ratio.

According to Figure 4a,b, the phenols extracted from the raspberry possessed high antioxidant activities and could effectively and rapidly inhibit the formation of DPPH and ABTS free radicals in solution. Furthermore, Boyne, at a concentration of 100 μg/mL, showed scavenging ratios up to 61.77% and 48.98% against DPPH and ABTS free radicals, respectively. The top five scavenging abilities of raspberry-extracted phenols against ABTS, from the highest to the lowest, were: Baojia, Meeker, Tulameen, Boyne, and Laguo. Additionally, the inhibition ratio against the DPPH free radical was significantly higher than that against the ABTS free radical, indicating that the scavenging ability of phenols was more favorable against lipo-soluble free radicals (DPPH) than that against hydro-soluble free radicals (ABTS).

#### 2.5.2. Scavenging Abilities Against Intracellular Free Radicals

The toxicities to HepG2 cells of the phenols extracted from five raspberry varieties, including Meeker, Boyne, Tulameen, Laguo, and Baojia, were evaluated at concentrations ranging from 20 to 100 μg/mL in order to confirm the safe doses for cell experiments. As shown in Figure 4c, all five raspberry-extracted phenols presented no significant effects on the survivability of the HepG2 cells at the concentrations tested (*p* < 0.05). The results also indicated that the concentration range could be utilized as safe doses for cell experiments.

As shown in Figure 4d, the scavenging abilities against intracellular ROS of the phenols extracted at concentrations ranging from 20–100 μg/mL from the raspberry varieties Meeker, Boyne, Tulameen, Laguo, and Baojia had dose–response relationships. Baoji-extracted phenols had the highest scavenging ability against ROS, with a ratio of 55.67%, followed by Meeker, Tulameen, and Boyne, in which the ratios were 53.73%, 46.83%, and 45.56%, respectively. The scavenging abilities against ROS of phenols from Meeker and Tulameen also showed significant dose–response relationships at low concentrations. However, when the concentration was higher than or equal to 60 μg/mL, the extracts displayed no significant increase in ROS scavenging capacity. The phenols extracted from Laguo had relatively weak ROS scavenging ability at concentrations ranging from 20 to 60 μg/mL; however, the ROS scavenging ability was enhanced to 24.52% when the concentration reached 80 μg/mL. On the basis of these results, it could be concluded that the antioxidant capacity was highest in the extracted phenols from Baojia, followed by Meeker, Tulameen, Boyne, and Laguo.

## 3. Experimental Conditions

### 3.1. Instruments

High-performance liquid chromatography (HPLC) analysis was performed using an Agilent1260 series HPLC system (Agilent Technologies Co. Ltd., Palo Alto, CA, USA), which was equipped with an online degasser (model G1322B, Agilent Technologies Co. Ltd., Palo Alto, CA, USA), a quaternary pump (model G1311C, Agilent Technologies Co. Ltd., Palo Alto, CA, USA), an autosampler (model G1329B, Agilent Technologies Co. Ltd., Palo Alto, CA, USA), a thermostat column compartment (model G1316B), and an ultraviolet detector (model G4212B, Agilent Technologies Co. Ltd., Palo Alto, CA, USA). The ultrasonic-assisted extraction of raspberry was conducted using an ultrasonic cleaner (KQ-500DE, Kunshan ultrasonic instrument Co., Kunshan, China). Absorbance was detected at 490 nm using a Multi-Mode Detection Platform (Molecular Devices, San Jose, CA, USA).

### 3.2. Materials and Reagents

Thirty-seven varieties of raspberry were identified by Professor Yourui Suo (Northwest Plateau Institute of Biology, Chinese Academy of Sciences) and were grown in the Qinghai-Tibetan plateau region. These raspberry varieties included 9 local varieties—Laguo, Cangjia, Baojia, Leren, Nanque, Ganchong, Pansheng, Layun, and Huazang—and 28 adventitious varieties—Meeker, Boyne, Tulameen, Fortodi, Lauren, Canby, Taylor, Tulameen, Reveille, Coho, Encore, Herokee, Kitsilano, Chilcotin, Titan, Latham, Raspberry Nano, Chillieack, Triple Crown, Boysenberry, Shawnee, Honey Queen, Full of Red Raspberry, Autumn Britten, Autumn Bliss, Heritage, Killarney, and Kiwigold.

Nine phenol standards, including gallic acid, catechin, chlorogenic acid, vanillic acid, syringic acid, cumaric acid, ferulic acid, rosemary acid, and quercetin, were of chromatographic grade and purchased from the Sigma Reagent Co. (St. Louis, MO, USA). Analytical graded chloroform, petroleum ether, and ethanol were obtained from the Shanghai Chemical Reagent Co. (Shanghai, China). Ultra-pure water was supplied by Watsons (Guangzhou, China). All other reagents used were of analytical grade unless otherwise stated.

### 3.3. Preparation of Standard Solutions

Stock solutions were first separately prepared in 90% acetonitrile. The stock solutions were then used to prepare the mixed standards (concentration of 1 × 10^−3^ mol/L) containing the nine phenols by diluting the corresponding stock solution with acetonitrile. Other diluted mixed standards were prepared by diluting the corresponding stock solutions with acetonitrile. All solutions were stored in a refrigerator (4 °C) until further use.

### 3.4. Sample Preparation

Preparation of analytical samples: Raspberry samples were dried in an electrical furnace at 60 °C until their weights were constant. The dried samples were then milled and kept at 4 °C until subsequent use. In the extraction of phenols, each sample was weighed to 50 mg in a brown ampere bottle and then dissolved with 2.0 mL of 65% ethanol. The sample was then ultra-sonicated at 50 °C for 50 min and centrifuged at 4000 rpm for 10 min. The supernatant was collected and filtered through a 0.22-μm nylon filter and then stored in a refrigerator (4 °C) until further analysis.

Preparation of antioxidant samples: In the extraction of phenols, each sample was weighed to 50 g and dissolved in 2000 mL of 65% ethanol. Volumes of the samples were first minimized to 100 mL in a vacuum at 40 °C. The samples were then loaded into a XAD-7 chromatographic column (4.0 cm × 60 cm, Yuwang Company, Shandong, China) and underwent adsorption for 1 h. The column was then eluted with acidified 1% ethanol (diluted in deionized water) at a flow rate of 2 mL/min to remove unbound and/or loosely bound non-phenol substances. The phenols were finally eluted using acidified absolute ethanol. The eluent was further concentrated in a vacuum at 40 °C, followed by freeze-drying to obtain phenol powders. A Sephadex LH20 glucan gel chromatographic column (1.8 cm × 100 cm, Yuwang Company, Shandong, China) was first equilibrated with phosphate-buffered saline (PBS) at pH 7.0. The extracted phenols weighed to 50 mg were eluted using PBS to remove phenols. The eluent was concentrated in a vacuum and freeze-dried to obtain the final extracted phenol samples. The samples were stored in the dark at 20 °C.

### 3.5. Experimental Design and Data Analysis

A three-variable and three-level Box–Behnken design (BBD) was applied to optimize the extraction process. Combined with the response surface methodology (RSM), BBD has the advantages of being efficient and simple, which can further provide interaction effects in the response values [35,36]. An adventitious variety, Meeker, was chosen to be a representative raspberry in the optimization of phenol extraction. The input variables and their values chosen in the optimization process were: X1, volume of extraction reagent (values = 1, 1.5, and 2 mL); X2, extraction time (values = 40, 50, and 60 min); and X3, extraction temperature (values = 35, 45, and 55 °C). The dependent variable (Y) was the peak area. The experimental designs for the extraction process are shown in Table 1. The experimental data were analyzed using Design Expert software (Version 7.1.6, Stat-Ease Inc., Minneapolis, MN, USA).

### 3.6. Biochemical Assays

Antioxidant activities of five widely grown raspberry varieties, including Meeker, Boyne, Tulameen, Laguo, and Baojia, were investigated.

#### 3.6.1. DPPH^−^ and ABTS^−^ Free Radical Scavenging Activity Assays

DPPH^−^ free radical scavenging activity [37,38]: DPPH solution was prepared in absolute ethanol at a concentration of 0.1 mmol/L and stored in the dark. A vitamin C (Vc) solution of 0.5 mg/mL was used as the reference. The freeze-dried raspberry phenols were diluted to various concentrations. Three types of equal-volume mixture solutions (2 mL each) were then prepared and measured for their absorbances to obtain parameters for the calculation of DPPH^−^ free radical scavenging activity: (1) A_sample_^1^ is the absorbance of samples and the DPPH solution mixture, which was prepared by first mixing the two components and then letting it stand in the dark for 30 min prior to measurement; (2) A_control_^1^ is the absorbance of the DPPH solution and solvent (i.e., distilled water or the corresponding buffer solutions) mixture; and (3) A_blank_^1^ is the absorbance of the testing sample and absolute ethanol mixture. The DPPH^−^ free radical scavenging activity was expressed as the scavenging ratio percentage, calculated using the following equation:

(2) $$\mathrm{Scavenging}\;\mathrm{ratio}\;\mathrm{of}\;\mathrm{DPPH}\;\left(\%\right)=\left[1-\left(A_{\mathrm{sample}}{}^{1}-A_{\mathrm{blank}}{}^{1}\right)÷A_{\mathrm{control}}{}^{1}\;\right]\times 100$$

ABTS^−^ free radical scavenging activity: Previous literature was taken as a reference [39], and absorbance values were measured on a spectrophotometer at 734 nm. First, the mother solution for the ABTS assay was prepared and stored in the dark for 12–16 h. Prior to use, the mother solution was diluted to appropriate concentrations using PBS and measured for the absorbance, which was deducted by the absorbance of the corresponding PBS blank control to obtain A_734_ = 0.7 (±0.02). To construct a standard curve, 10 mmol/L Trolox standard solution (Sigma Reagent Co., St. Louis, MO, USA) was first diluted to various concentrations. The standard curve was then plotted according to the scavenging ratio to ABTS [40]. The antioxidant ability with Trolox equivalency was calculated using the following equation:

(3) $$\mathrm{ABTS}\;\mathrm{scavenging}\;\mathrm{ratio}\;\left(\%\right)=\left[1-\left(A_{\mathrm{sample}}{}^{2}-A_{\mathrm{blank}}{}^{2}\right)÷A_{\mathrm{control}}{}^{2}\right]\times 100\%$$

where A_sample_^2^ is the absorbance of the testing sample, A_blank_^2^ is the sample background absorbance, and A_control_^2^ is the absorbance when the sample is not present (i.e., buffer alone).

#### 3.6.2. Assays of the Intracellular Activities of Phenols

Cell preparation: HepG2 cells were cultured in Dulbecco’s modified eagle medium (DMEM) containing 10% fetal calf serum. Cell passages were conducted when the cell density achieved 80–90%, and stable and well-grown cells were used for further experiments. Cells at log-phase were seeded in a 96-well plate for subsequent experiments.

Cell toxicity assay: cellular toxicological evaluation was carried out using an MTT (3-(4,5-dimethylthiazol-2-yl)-2,5-diphenyltetrazolium bromide) assay. Cells were first seeded in 96-well plates at a cell density of 8 × 10^4^ cells/mL and cultured at 37 °C for 24 h. After that, the culture medium was replaced with the incomplete culture medium supplemented with various concentrations of phenols, and it was further cultured at the same temperature for 24 h. Thereafter, the fluid was removed, and 200 μL of incomplete culture medium containing MTT solution was added to obtain a final concentration of 0.5 mg/mL, which continued to incubate at 37 °C. After 4 h of incubation, the fluid was removed, and 150 μL of dimethyl sulfoxide (DMSO) was added. The plate was placed on a shaker running at low speed for 5 min to allow for mixing of the cells and DMSO. Finally, a Multi-Mode Detection Platform was employed, and the absorbance at 570 nm was measured.

Intracellular ROS detection: HepG2 Cells were seeded in 96-well plates at a cell density of 8 × 10^4^ cells/mL (200 μL/well) and incubated under 5% CO_2_ at 37 °C for 24 h. After 24 h, the medium was carefully removed, and the cells were then incubated in DMEM medium, without serum, supplemented with phenol samples under 5% CO_2_ at 37 °C for 24 h. This was followed by the addition of 200 μL of HBSS (Hank’s buffered salt solution) containing 25 μmol/L of 2′-7′- dichlorodihydrofluorescein diacetate (DCFH-DA) and was further incubated for 1 h in the same conditions. After that, the fluid was removed, and the cells were washed three times with HBSS solution. The cells were then treated with 100 μL of 0.6 mol/L AAPH (2,2′-azobis(2-amidinopropane) dihydrochloride) and incubated for 30 min in the CO_2_ incubator in the same conditions. The Multi-Mode Detection Platform was finally employed, and fluorescence intensities were measured at the excitation and emission wavelengths of 485 and 530 nm, respectively.

## 4. Conclusions

In this work, an efficient method was developed for the rapid extraction and separation of nine phenols, including gallic acid, catechin, chlorogenic acid, vanillic acid, syringic acid, cumaric acid, ferulic acid, rosemary acid, and quercetin, from 37 different varieties of raspberry and for antioxidative activities research. The analysis of phenol contents from various raspberry varieties indicated that the exotic varieties Baojia, Meeker, Tulameen, and Boyne had significantly higher phenol contents than local varieties, such as Laguo. In turn, these varieties possessed higher antioxidant activities, as demonstrated in the results from scavenging activities against DPPH and ABTS free radicals and from intracellular ROS experiments. This is the first report on the quantitative analyses of 9 phenolic compounds and their antioxidant activities from 37 varieties of raspberry grown in the Qinghai-Tibetan Plateau region. On this basis, this research provides a theoretical framework for the various choices of raspberry suitable for product development in the Qinghai-Tibetan Plateau region.

## Figures and Tables

**Table 1:** Experimental design and data for the best extraction conditions obtained from BBD for phenolic acids (*n* = 3).

Run	Volume of Extraction Reagent (mL)	Time of Extraction (min)	Temperature of Extraction (°C)	Peak Area
1	1.50	40.00	55.00	5097.98
2	1.50	50.00	45.00	5153.31
3	1.00	60.00	45.00	5011.27
4	2.00	50.00	35.00	5028.35
5	1.50	60.00	55.00	5089.75
6	1.50	50.00	45.00	5167.35
7	1.00	40.00	45.00	5009.34
8	1.50	50.00	45.00	5145.27
9	2.00	40.00	45.00	5027.78
10	1.50	60.00	35.00	5027.31
11	2.00	50.00	55.00	5098.31
12	1.00	50.00	35.00	5010.68
13	1.50	50.00	45.00	5149.74
14	1.00	50.00	55.00	5012.35
15	1.50	40.00	35.00	5018.39
16	1.50	50.00	45.00	5154.41
17	2.00	60.00	45.00	5113.34

**Table 2:** Linear regression equation, correlation coefficients, limits of detection (LODs), limits of quantification (LOQs), reproducibility of retention time and peak area, and intra- and inter-day precisions.

Analyte	Regression Equation	r	LOD (µg/L)	LOQ (µg/L)	Instrument Precision (*n* = 6)		Method Precision (*n* = 3)	
					Intra-Day	Inter-Day	Intra-Day	Inter-Day
Gallic acid	y = 0.867x − 0.039	0.9987	0.32	1.02	0.6	1.1	1.5	3.1
Catechin	y = 3.925x − 0.280	0.9962	0.30	0.97	0.6	1.0	1.3	2.5
Chlorogenic acid	y = 1.790x − 0.029	0.9973	0.32	0.99	0.8	1.3	1.7	3.5
vanillic acid	y = 3.993x − 0.061	0.9985	0.27	0.81	0.7	0.9	1.4	2.7
Syringic acid	y = 6.977x − 0.155	0.9976	0.25	0.79	0.8	1.2	1.6	3.3
Cumaric acid	y = 10.132x − 0.139	0.9968	0.18	0.57	1.1	1.4	1.7	3.4
Ferulic acid	y = 7.101x − 0.073	0.9963	0.23	0.69	0.7	1.1	1.4	2.9
Rosemary acid	y = 0.657x − 0.002	0.9972	0.32	0.98	0.5	0.8	1.2	2.3
Quercetin acid	y = 7.023x − 0.061	0.9977	0.11	0.35	0.7	1.0	1.5	3.3

**Table 3:** Recovery studies of the proposed method at three concentration levels.

Analyte	Concentration 1			Concentration 2			Concentration 3		
	Added	Found	Recovery	Added	Found	Recovery	Added	Found	Recovery
	(µg/L)	(µg/L)	(%)	(µg/L)	(µg/L)	(%)	(µg/L)	(µg/L)	(%)
Gallic acid	0.5	0.51	102	1.0	0.97	97	2.0	2.01	100.5
Catechin	0.5	0.49	98	1.0	0.99	99	2.0	2.00	100
Chlorogenic acid	0.5	0.49	98	1.0	1.00	100	2.0	1.97	98.5
Vanillic acid	0.5	0.50	100	1.0	1.01	101	2.0	1.99	99.5
Syringic acid	0.5	0.47	94	1.0	0.97	97	2.0	1.97	98.5
Cumaric acid	0.5	0.48	96	1.0	0.99	99	2.0	2.02	101.1
Ferulic acid	0.5	0.49	98	1.0	0.96	96	2.0	1.98	99.3
Rosemary acid	0.5	0.50	100	1.0	0.98	98	2.0	2.00	100
Quercetin acid	0.5	0.51	102	1.0	1.02	102	2.0	1.99	99.5

**Table 4:** Main phenolic acid contents in *Rubus idaeus L.* (mean ± SD).

Samples	Gallic Acid	Catechin	Chlorogenic Acid	Vanillic Acid	Syringic Acid	Cumaric Acid	Ferulic Acid	Rosemary Acid	Quercetin Acid
	(mg/g, *n* = 3)	(mg/g, *n* = 3)	(mg/g, *n* = 3)	(mg/g, *n* = 3)	(mg/g, *n* = 3)	(mg/g, *n* = 3)	(mg/g, *n* = 3)	(mg/g, *n* = 3)	(mg/g, *n* = 3)
Meeker	145.87 ± 0.63	30.57 ± 0.89	77.00 ± 0.68	-	189.30 ± 0.28	198.21 ± 0.67	-	10.92 ± 0.22	-
Boyne	36.74 ± 0.42	9.91 ± 0.40	20.37 ± 0.46	5.03 ± 0.74	-	26.89 ± 0.75	-	21.43 ± 0.46	5.60 ± 0.44
Tulameen	77.74 ± 0.97	2.39 ± 0.81	10.98 ± 0.84	-	-	174.25 ± 0.27	2.97 ± 0.68	2.89 ± 0.58	-
Fortodi	10.13 ± 0.64	-	22.43 ± 0.21	6.69 ± 0.47	-	58.57 ± 0.61	-	21.95 ± 0.26	-
Lauren	9.92 ± 0.32	-	16.46 ± 0.25	-	17.67 ± 0.39	-	2.45 ± 0.45	3.65 ± 0.84	-
Canby	9.56 ± 0.35	-	2.27 ± 0.42	-	91.78 ± 0.26	-	-	-	-
Taylor	8.79 ± 0.73	1.03 ± 0.09	3.72 ± 0.95	-	47.25 ± 0.87	-	-	11.14 ± 0.78	-
Tulameen	195.51 ± 0.12	28.09 ± 0.95	46.48 ± 0.41	-	-	8.52 ± 0.93	-	25.06 ± 0.87	-
Reveille	24.24 ± 0.81	2.42 ± 0.99	11.84 ± 0.31	-	-	-	-	12.66 ± 0.31	-
Coho	115.89 ± 0.71	59.69 ± 0.89	-	-	-	-	-	14.68 ± 0.07	-
Encore	22.53 ± 0.85	2.24 ± 0.99	11.98 ± 0.30	2.24 ± 0.59	-	-	-	38.55 ± 0.75	-
Herokee	21.25 ± 0.12	1.52 ± 0.34	11.88 ± 0.70	-	-	-	-	10.89 ± 0.55	-
Kitsilano	113.22 ± 0.76	9.43 ± 0.84	27.96 ± 0.19	-	2.10 ± 0.09	26.14 ± 0.39	2.79 ± 0.84	11.06 ± 0.38	-
Chilcotin	70.25 ± 0.93	3.67 ± 0.76	13.98 ± 0.84	-	-	-	-	-	-
Titan	113.61 ± 0.44	38.26 ± 0.50	68.79 ± 0.31	-	-	14.95 ± 0.63	-	5.43 ± 0.68	-
Latham	23.63 ± 0.84	3.25 ± 0.18	13.00 ± 0.56	-	-	10.24 ± 0.15	-	7.53 ± 0.93	-
Raspberry Nano	41.28 ± 0.84	5.12 ± 0.33	10.04 ± 0.28	-	-	-	-	4.77 ± 0.57	-
Chillieack	-	29.56 ± 0.77	54.63 ± 0.62	-	2.71 ± 0.54	23.73 ± 0.88	2.14 ± 0.38	8.18 ± 0.35	-
Triple Crown	-	5.88 ± 0.22	30.56 ± 0.99	-	-	12.33 ± 0.54	-	7.87 ± 0.76	-
Boysenberry	-	-	20.04 ± 0.39	-	-	-	-	33.62 ± 0.86	-
Shawnee	-	2.29 ± 0.79	391.60 ± 0.48	-	56.01 ± 0.27	-	-	7.43 ± 0.43	-
Honey Queen	81.50 ± 0.15	8.83 ± 0.83	28.34 ± 0.83	-	33.87 ± 0.848	-	-	6.57 ± 0.12	-
Full of red Raspberry	93.39 ± 0.17	13.45 ± 0.22	57.85 ± 0.21	-	313.78 ± 0.39	6.23 ± 0.76	-	71.16 ± 0.61	-
Autumn Britten	6.44 ± 0.67	-	13.45 ± 0.55	2.33 ± 0.57	22.44 ± 0.39	-	-	23.50 ± 0.19	-
Autumn Bliss	47.89 ± 0.21	4.49 ± 0.48	10.46 ± 0.25	-	-	27.60 ± 0.91	2.25 ± 0.98	10.97 ± 0.24	-
Heritage	13.76 ± 0.18	-	18.28 ± 0.39	-	-	-	-	14.89 ± 0.59	-
Killarney	14.40 ± 0.24	2.41 ± 0.86	5.07 ± 0.74	-	-	16.17 ± 0.41	-	5.05 ± 0.64	-
Kiwigold	17.46 ± 0.37	2.15 ± 0.16	80.68 ± 0.41	-	-	-	-	5.14 ± 0.28	-
Laguo	-	46.84 ± 0.14	336.69 ± 0.83	-	-	-	-	23.74 ± 0.35	-
Cangjia	-	3.49 ± 0.48	-	-	-	-	5.81 ± 0.32	6.93 ± 0.95	-
Baojia	-	41.99 ± 0.78	-	-	-	-	-	8.33 ± 0.12	-
Leren	-	10.49 ± 0.61	-	-	-	-	-	3.71 ± 0.98	-
Nanque	11.09 ± 0.52	-	3.18 ± 0.11	14.65 ± 0.39	-	2.60 ± 0.34	3.37 ± 0.87	6.24 ± 0.79	-
Ganchong	64.17 ± 0.76	38.58 ± 0.72	248.13 ± 0.18	-	-	-	-	10.57 ± 0.22	-
Pansheng	70.63 ± 0.96	21.54 ± 0.36	44.30 ± 0.38	-	2.36 ± 0.49	7.24 ± 0.79	2.84 ± 0.88	21.18 ± 0.75	-
Layun	20.55 ± 0.92	-	37.69 ± 0.79	5.71 ± 0.27	-	11.46 ± 0.31	-	27.61 ± 0.78	-
Huazang	10.81 ± 0.14	-	7.19 ± 0.31	-	-	-	-	11.93 ± 0.79	-

^1^ Data are expressed as mean value ± S.D. - signifies not detected.

**Table 5:** Total phenolic acid contents in *Rubus idaeus L.* growing in different regions (mean ± SD) (mg/100g).

Samples	Qinghai-Tibetan Plateau	Northern Greece [26]	Spain [27]	Turkey [24]	Belgrade [28]	Norway [29]	Brazil [30]	NewYork [31,32]	Bursa [33]	Lithuanian [5]	Finland [34]
Heritage	2715.36 ± 36.77	1905 ± 58	1232.28 ± 66.49	3064.64 ± 51.07	-	297.7	446.79	512.7 ± 4.7	1463.7 ± 22.8	-	317 ± 5
Meeker	5914.37 ± 73.86	2116 ± 44	-	-	-	-	-	444	-	388.8 ± 11.3	-
Autumn Bliss	1977.74 ± 21.18	2494 ± 77	1364.32 ± 80.14	-	372 ± 14	-	553.23	396	-	-	-
Taylor	2136.16 ± 28.12	1891 ± 76	-	-	-	-	-	-	-	-	-
Boysenberry	678.68 ± 10.03	-	-	-	-	-	319.75		-	-	-
Kiwigold	1066.78 ± 12.18	-	-	-	-	-	-	451.1 ± 4.5	-	-	-
AutumnBritten	896.58 ± 14.30	-	-	-	-	-	-	367	-	-	-
Boyne	715.26 ± 11.16	-	-	-	-	-	-	386	-	-	-
Tulameen	1489.63 ± 17.52	-	-	-	-	-	-	386	-	-	-
Coho	1840.76 ± 17.30	-	-	-	-	-	-	383	-	-	-

^1.^ -Not tested.
